# Multifaceted Structurally Coloured Materials: Diffraction and Total Internal Reflection (TIR) from Nanoscale Surface Wrinkling

**DOI:** 10.3390/molecules28041710

**Published:** 2023-02-10

**Authors:** Annabelle Tan, Zain Ahmad, Pete Vukusic, João T. Cabral

**Affiliations:** 1 Department of Chemical Engineering, Imperial College London, London SW7 2AZ, UK; 2 Centre for Processable Electronics, Imperial College London, London SW7 2AZ, UK; 3 School of Physics, University of Exeter, Stocker Road, Exeter EX4 4QL, UK

**Keywords:** structural colour, multifaceted, diffraction, total internal reflection (TIR), polydimethylsiloxane (PDMS), wrinkling, plasma oxidation, multiaxial, polyhedra, GRISM

## Abstract

We investigate the combined effects of surface diffraction and total internal reflection (TIR) in the design of 3-dimensional materials exhibiting distinct structural colour on various facets. We employ mechanical wrinkling to introduce surface diffraction gratings (from the nano to the micron scales) on one face of an elastomeric rectangular parallelepiped-shaped slab and explore the roles, in the perceived colours, of wrinkling pattern, wavelength, the directionality of incident light and observation angles. We propose a simple model that satisfactorily accounts for all experimental observations. Employing polydimethylsiloxane (PDMS), which readily swells in the presence of various liquids and gases, we demonstrate that such multifaceted colours can respond to their environment. By coupling a right angle triangular prism with a surface grating, we demonstrate the straightforward fabrication of a so-called GRISM (GRating + prISM). Finally, using a range of examples, we outline possibilities for a predictive material design using multi-axial wrinkling patterns and more complex polyhedra.

## 1. Introduction

Structural colour abounds in nature, in both the animal and plant kingdoms, emerging from microscopically structured surfaces and bulk materials, able to cause visible light interference [1,2,3,4,5,6,7], with or without the presence of chemical pigments. During the past two decades or so, a range of bioinspired synthetic and processing strategies have been proposed to engineer structural colour on surfaces and bulk materials. These include multilayer film lamination [8,9], the assembly of photonic crystals [10,11] and metasurfaces [12,13], whose colour can be static or respond to external stimuli [14,15,16,17]. Recently, novel structural coloured films and microscale concave interfaces based on total internal reflection (TIR) interference have also been reported, combining the effects of thin-film interference and TIR [18,19,20]. A number of practical applications of such materials have been reported, where structural colour sensors and devices based on responsive soft materials have been fabricated [21,22,23,24].

Surface topography and, specifically, undulations caused by buckling or wrinkling, are found in a range of flowers and insects [25,26,27,28,29], where these wrinkled surfaces, effectively acting as diffraction gratings, can yield brilliant structural colours which depend on pattern periodicity *d* and sufficiently large pattern amplitudes, and observation angle. Pattern orientation, from uni-directional to isotropic (or random), can further restrict or modulate the viewable angles of the perceived structural colour [30,31]. When white light impinges onto a wrinkled surface, it can diffract in transmission or reflection, or both, and propagate further. While the combination of TIR and light diffraction has been reported in the characterisation of fluids in microfluidic cells [32] and in the measurement of the refractive index of liquids [33], to the best of our knowledge, their combination has not been exploited in the design and fabrication of multi-faceted materials exhibiting structural colour. Naturally occurring materials exhibiting structural colour generally often exhibit colour and colour modulations across multiple viewing angles. By contrast, colour generated from surface diffraction is directional, even in isotropic or multiaxial diffractive surfaces [30,31], contrasting with the appearance of bulk photonic and anisotropic structures. We therefore explore the feasibility of designing multi-faceted and modulated structural colour through the combination of wrinkling and diffraction and TIR selection and propagation. We expect that such multi-faceted structurally coloured materials can approximate more closely ‘bulk’ structural colours found in nature [34].

**Figure 1:**
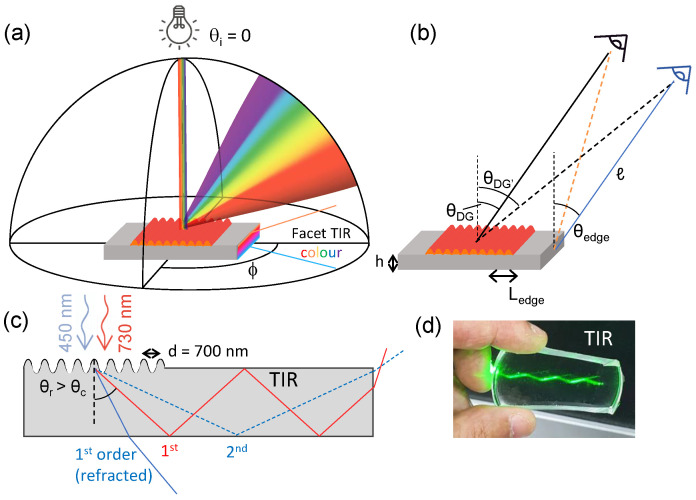
(**a**) Schematic of light diffraction from a 1D wrinkled surface, with normal incident light, and total internal reflection (TIR) leading to light propagation onto the sample facet. (**b**) Experimental geometry, defining different observation angles $\theta_{DG}$, defined from the normal of the diffraction grating; $\theta_{edge}$, defined from the normal of the sample edge, or facet; $\theta_{DG^{\prime }}$, defined for the “edge observer” from the normal of the diffraction grating when $\theta_{edge}$ = $\theta_{DG}$. The lines from the facet (orange and blue) correspond to a (fixed) sample-observer distance of *ℓ*. (**c**) Cross-sectional schematic of TIR propagation, illustrated for two incident wavelengths (λ = 450 and 730 nm) and surface periodicity *d* = 700 nm: for the shorter λ, the 1st diffraction order is below the critical angle on the sample’s bottom surface and is thus refracted out of the sample, while the 2nd order propagates by TIR and exits at the sample edge; the 1st diffraction order of the longer λ undergoes TIR thus also contributes to the edge colour. (**d**) Visualisation of TIR within a 5 mm thick PDMS coupon, with a monochromatic beam (laser λ = 533 nm) at a high incident angle.

Visible light diffraction through reflection can lead to the emergence of structural colour from patterned surfaces, of appropriate periodicity and amplitude, which varies with observation angle, as illustrated in Figure 1. Evidently, the surface can also act as a transmission grating where the light diffracts *into* the sample. If the material properties support TIR, we envisage that structural colour can be indirectly observed at the facet (or facets) of the material, which we term here “facet TIR colour” (Figure 1a). However, light diffraction will generate a distribution of wavelengths at different angles, and several diffractions of varying intensities; therefore, such colour may differ from the original diffraction spectrum owing to TIR propagation rules. With structural colour observable from two (or more) different viewing perspectives for such a material, we introduce the different nomenclatures for the observation angles with respect to the surface normal, one associated with the surface, $\theta_{DG}$, and the other with the facet, $\theta_{edge}$. The offset angle when viewing the surface with respect to $\theta_{DG}$ is termed $\theta_{DG^{\prime }}$, which depends on the length of unwrinkled material $L_{edge}$ (Figure 1b). Figure 1c depicts the cross-sectional profile of light diffracting into the medium, and propagating through the medium, by total internal reflection, toward a facet (Figure 1c). Ray tracing for two wavelengths, 450 and 730 nm, is shown, to illustrate the colour and diffraction order selection, indicating that different colours to those diffracted by the surface pattern may be expected at the material facets. Figure 1d demonstrates the TIR of monochromatic light (λ = 533 nm laser) within a slab of PDMS.

Soft materials, such as elastomers, are advantageous in the fabrication of stiff-soft bilayers and wrinkled surfaces, and mechanical strain can readily tune the surface periodicity and amplitude. Typically, bilayers are fabricated through the deposition or lamination of a thin and stiff film on a soft substrate such as polydimethylsiloxane (PDMS). Mismatches between the mechanical properties of two films, provided they adhere strongly together (to minimize delamination, cracking, etc.), result in surface buckling under strain, which can be induced commonly through mechanical deformation, through thermal expansion/contraction, volume changes, etc. Plasma oxidation of PDMS provides a convenient route to generating a glassy (SiOx) thin film atop the PDMS surface [35], inducing a mismatch in elastic moduli between the thin skin and the bulk. Further, it enables the precise control of film thickness growth [36,37,38,39,40], and the fabrication of wrinkled surfaces with varying periodicity (*d*) and amplitude (A), that can range from the nm scale to several 100s μm. Permanent wrinkles can be formed when the plasma exposure is carried out with the PDMS coupon under pre-strained conditions, while transient wrinkles can be excited on otherwise planar surfaces upon the application of strain on a bilayer fabricated at rest. Transparent elastomers, such as PDMS, are well suited for optical devices, and the design and fabrication of PDMS sinusoidal phase gratings, with tunable periodicity and amplitude, via plasma-oxidation, has been previously demonstrated, resulting in structural colour and mechanochromic response [17,31,41,42,43,44] for a judicious choice of system parameters (skin thickness, mechanical moduli, strain, etc.).

Building upon previous work on reflective diffraction gratings fabricated by surface wrinkling, we consider the possible roles of light transmission/refraction, in the design of materials exhibiting structural colour on various facets. Specifically, we seek to establish and model the conditions and limits for total internal reflection (TIR), wavelength and diffraction order selection, the roles played by the geometry of the surface grating, as well as overall polyhedral shape and environmental conditions, on the resulting colour.

## 2. Results and Discussion

### 2.1. Structural Colour of Wrinkled Surfaces through Surface Diffraction

**Figure 2:**
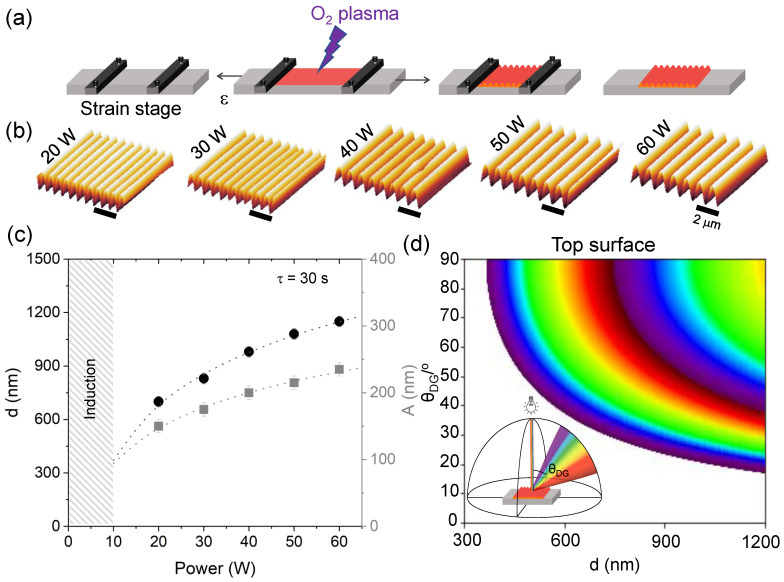
(**a**) Schematic of the fabrication of a 1D wrinkled sample: a PDMS elastomer coupon is mechanically strained and then exposed to oxygen plasma. Upon strain relaxation, a wrinkled surface remains (at rest conditions). (**b**) AFM scans of samples fabricated at different plasma powers (*P* = 20 W to 60 W) and fixed exposure time τ = 30 s, and pre-strain ϵ = 0.5; the scale bar corresponds to 2 μm. (**c**) Wrinkling periodicity (*d*) and amplitude (*A*) measured for the samples above; the shaded area corresponds to an induction stage for glassy skin and wrinkling onset. (**d**) Structural colour map computed for incident white light at $\theta_{i}$ = 0, surface periodicity 300 ≤d≤ 1200 nm, and observation angle 0 $\leq \theta_{DG}\leq$ 90${}^{\circ }$, considering the first two diffraction orders (adapted from Ref. [31]). At lower *d* (≲380 nm), UV can take place.

We have fabricated a range of one-dimensional (1D) wrinkled structures by applying uni-axial mechanical strain (ϵ = 0.5) on PDMS coupons, subsequently exposed to oxygen plasma at varying power (20–60 W). Upon relaxation of strain, the bilayer yields a sinusoidal profile, at sufficiently low deformations (Figure 2a). Wrinkles of different periodicities are readily obtained from the variation in plasma power (at constant exposure time), as shown by the atomic force microscopy (AFM) profiles in Figure 2b. The associated periodicity *d* and amplitude *A* can be expressed as [45,46],

(1) $$d=\frac{2\pi h{\left({\bar{E}}_{f}/\left(3{\bar{E}}_{s}\right)\right)}^{\frac{1}{3}}}{\left(1+\epsilon \right){\left(1+\xi \right)}^{\frac{1}{3}}}$$

(2) $$A=\frac{h{\left(\epsilon /\epsilon_{c}-1\right)}^{\frac{1}{2}}}{{\left(1+\epsilon \right)}^{\frac{1}{2}}{\left(1+\xi \right)}^{\frac{1}{3}}}$$

where *h* is the converted film thickness, ${\bar{E}}_{f}$ and ${\bar{E}}_{s}$ are the in-plane strain moduli of the film and substrate, respectively, given by $\bar{E}=E/\left(1-\nu^{2}\right)$, where *E* is Young’s modulus and ν the Poisson ratio (≃0.5 for PDMS); ξ=5ϵ(1+ϵ)/32, accounting for the nonlinearity of the stress–strain relationship of the substrate in the finite deformation regime (i.e., non-Hookean response). Here, we refer to the surface periodicity as *d*, instead of the customary surface wavelength λ, to avoid confusion with the wavelength of light. In order to trigger the mechanical instability, a certain “critical” strain $\epsilon_{c}$ must be exceeded:

(3) $$\epsilon_{c}=\frac{1}{4}{\left(\frac{3{\bar{E}}_{s}}{{\bar{E}}_{f}}\right)}^{\frac{2}{3}}$$

The experimentally measured logarithmic dependence of periodicity and amplitude with plasma exposure power (20 to 60 W), at a fixed exposure time τ = 30 s, is shown in Figure 2c. This dependence is attributed to the mechanisms and kinetics of the frontal growth and propagation of the glassy skin layer [37,38]. With this range of conditions, wrinkle periodicities from 700 nm to 1150 nm can be readily obtained.

In previous literature, we [31] and others [42,43,44] have demonstrated that wrinkling by plasma oxidation of PDMS provides an effective means of fabricating surfaces with structural colour, via the light diffraction on the surface grating. Control of colour brightness, hue, and viewable-angle mechanochromism were demonstrated. The approach is attractive due to the versatility of the fabrication method, where the wrinkling profile (*d*, *A*) can be tuned via plasma exposure conditions (defining *h* and moduli) and/or applied strain. The behaviour of the diffraction gratings can be described by the general form of the diffraction equation,

(4) $$m\lambda =d(\text{sin}\theta_{i}+\text{sin}\theta_{DG})$$

where integer *m* is the diffraction order of light of wavelength λ. From Equation (4), we compute the wavelength of light diffracted by wrinkles of varying periodicities across a range of detection angles, from 0${}^{\circ }$ to 90${}^{\circ }$. Figure 1d shows the expected light diffraction at each $\theta_{DG}$ for wrinkled surfaces with periodicities up to *d* = 1200 nm, when the incident light is normal to the surface ($\theta_{i}$ = 0${}^{\circ }$). When the periodicity of the wrinkles is shorter than that of the wavelength of visible light, no colour will be observed at the surface. At this range of *d*, discrete structural colours can be observed since the grating diffracts with no colour mixing of different orders involved.

### 2.2. Total Internal Reflection (TIR) and Selection of Facet Colour

**Figure 3:**
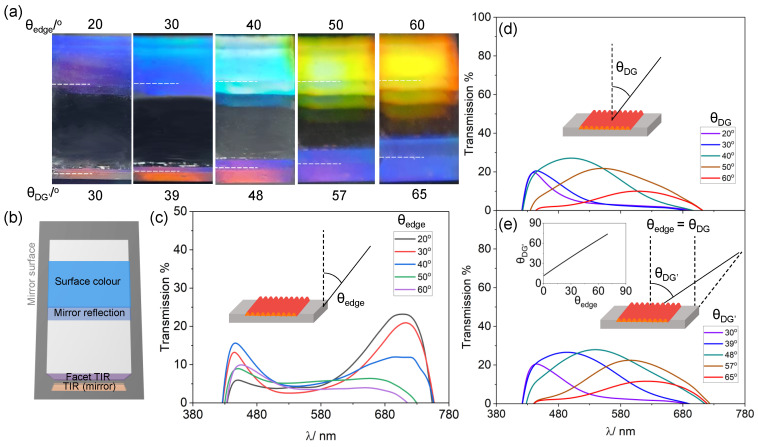
(**a**) Optical image of the structural colour of a 1D surface wrinkled sample with *d* = 700 nm (P = 20 W, τ = 30 s, ϵ = 0.5): reflective diffraction (top) and TIR (facet) lead to distinct colours at fixed observation angles. (**b**) Experimental setup depicting the sample support on a mirror (reflecting also the downward refracted light) the dashed line in (**a**) demarcates the lower sample edge. (**c**) Spectroscopic measurements of (TIR) structural colour at the facet at varying $\theta_{edge}$. (**d**) Spectra of reflected diffracted light, measured as a function of $\theta_{DG}$, and (**e**) $\theta_{DG^{\prime }}$, the offset in observation angle when $\theta_{edge}$ = $\theta_{DG}$, defined in Figure 1, and related to $\theta_{edge}$, are shown in the inset.

Similarly to surface structural colour derived from the diffraction of light in reflection from a wrinkled surface, the observation of facet TIR colour is also expected to be angle-dependent. Light diffracted in transmission can be further propagated via TIR, under specific conditions, resulting in structural colour (indirectly) appearing on the facets of transparent materials. Figure 3a shows a series of optical images, taken at varying observation angles, of a sample with surface grating periodicity *d* = 700 nm (fabricated with plasma conditions P = 20 W, τ = 30 s, ε = 0.5), that exhibits a range of structural colours. For clarity, the sample was placed on a mirror that acts as a reflective substrate and was observed at $\theta_{edge}$ at 10${}^{\circ }$ intervals of observation angle, from 20 to 60${}^{\circ }$. Concurrently, the surface structural colour can also be viewed, but with an offset angle of $\theta_{DG^{\prime }}$ ranging from 30 to 65${}^{\circ }$ when *l* = 10 cm and $L_{edge}$ = 2 cm. The white line on the optical images indicates the boundaries between facet colour and the mirror reflection. Figure 3b shows a schematic of the sample set-up, including the role of the “TIR (mirror)” where refracted rays from the facet impinging onto the mirror result in an additional colour perceived (which can be different from the facet colour).

As $\theta_{edge}$ increases, the facet colour transitions from purple/red to dark blue while the surface colour is red-shifted from blue to orange. Transmission measurements were taken with respect to both facet and surface. The associated spectroscopy measurements at the facet are shown in Figure 3, indicating that the reflectance peak shifts from the red region (≈730 nm) to the blue region (≈450–480 nm). For surface structural colour, 2 different sets of spectroscopy measurements are represented: Figure 3d shows the spectra when the measurements are taken with respect to $\theta_{DG}$, where $\theta_{DG}$ = $\theta_{edge}$ while Figure 3e show measurements taken at $\theta_{DG^{\prime }}$, where the angle is offset with respect to $\theta_{edge}$ (relationship shown in inset). The transmission spectra are recorded and then normalised for background and incident light intensities (Figure S1). Both spectra show that, as the observation angle increases, the measured spectra are red-shifted as the peak shifts from the blue region at λ ≈ 450 nm to λ ≈ 650 nm.

#### A Minimal Model for Facet Colour: TIR and Incident Light Dispersion

**Figure 4:**
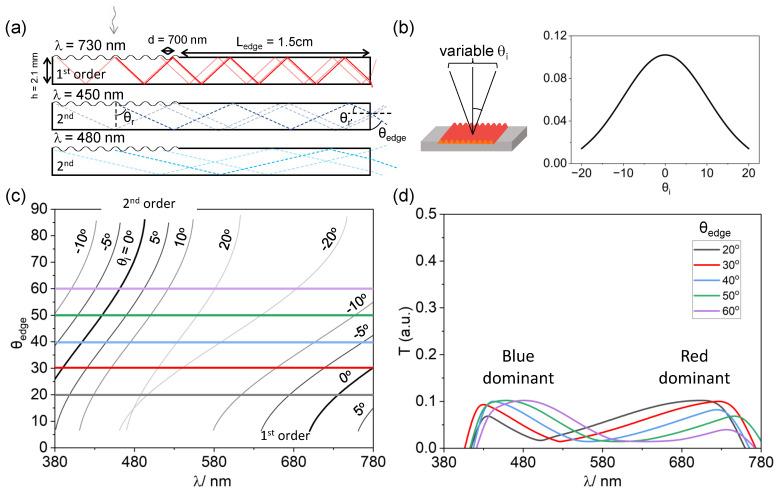
(**a**) Schematic diagram representing the modelling calculations, for a given coupon dimensions (height *h*, length $L_{DG}+L_{edge}$) and periodicity *d*, each individual wavelength is ray-traced, diffraction orders that do not meet TIR conditions are excluded, and the refracted colours at the edge computed as a function of $\theta_{edge}$. (**b**) Schematic depicting the distribution of incident angles $\theta_{i}$ onto a point in the sample and illustrative of angular dispersion, described by a Gaussian profile with a half-width at half-maximum (HWHM) of 10${}^{\circ }$. (**c**) Computed relation between ($\theta_{edge}$) and λ at fixed $\theta_{i}$ for the 1st and 2nd diffraction orders. Representative fixed $\theta_{edge}$ angles are shown by the horizontal lines. (**d**) Predicted spectra for the edge colour, assuming the Gaussian distribution shown in (**b**), and accounting for the light source λ distribution (Figure S1). At this *d*, the edge colour is expected to change from red-dominant to blue-dominant upon increasing $\theta_{edge}$, in agreement with Figure 3a,c.

The resulting structural colour at the facet can be modelled using the principles of TIR within a medium. The angular dispersion of the incident white light from a flood-illuminating source is also included in the model, to reflect practically-relevant conditions. We first describe the behaviour of a sample with a surface diffraction grating of periodicity *d* = 700 nm, depicted in Figure 3. Figure 4a is a schematic diagram showing three selected wavelengths, λ = 450, 480 and 730 nm, of light incident on the diffracting surface of the sample. These wavelengths were chosen due to their peak positions in the transmission measurements. We first consider that the rays are incident on the grating where $\theta_{i}$ = 0${}^{\circ }$. Within the medium, the general diffraction equation can be modified for a transmission diffraction grating to take into account the respective refractive index ($n_{0}$ in air, $n_{1}$ in PDMS):

(5) $$m\lambda =d(n_{0}\text{sin}\theta_{i}+n_{1}\text{sin}\theta_{r})$$

where $\theta_{r}$ denotes the angle at which light is diffracted into the sample. λ = 450 nm diffracts at 27.1${}^{\circ }$ (1st order) and 65.8${}^{\circ }$ (2nd order), λ = 480 nm at 29.1${}^{\circ }$ (1st order) and 76.6${}^{\circ }$ (2nd order), and λ = 730 nm at 45.17${}^{\circ }$ (1st order). When the angle of light in the medium (with respect to the normal) exceeds the critical angle, the light undergoes total internal reflection. The critical angle within PDMS, taking into account the refractive index of air ($n_{0}$ = 1) and of PDMS ($n_{1}≃$ 1.41) is estimated to be

(6) $$\theta_{c}=\arcsin\left(\frac{n_{0}}{n_{1}}\right)=45.17^{\circ }$$

As the 1st order of 450 nm and 480 nm wavelengths does not exceed the critical angle, the ray of light refracts out of the sample without being reflected internally when reaching the boundary of the sample. On the other hand, as the angle of diffraction for the 2nd order of λ = 450 nm and 480 nm and 1st order of 730 nm exceeds that of the critical angle for the system, these undergo TIR and propagate through the sample before exiting at the edge we term the “facet” with an angle of $\theta_{r^{\prime }}$, with respect to the (horizontal) facet normal, where $\theta_{r^{\prime }}$ = 90${}^{\circ }$−$\theta_{r}$. From there, we can calculate $\theta_{edge}$ from Equation (7), which is the complementary angle to that of the ray refracted at the facet:

(7) $$\theta_{edge}=90^{\circ }-\arcsin\left(n_{1}\times \sin\theta_{r^{\prime }}\right)$$

Refracted rays at the facet can either exit towards the mirror (below the sample) or the observer. Due to the size of the incident beam spot, light is incident across the entirety of the grating. As a result, it is insufficient to consider the pathway of only a singular ray incident on the grating, but it is also important to consider incidence at different points along the wrinkles (transparent lines). By considering this, it offers a better understanding of the colours emerging at the facet, thereby leaving the sample at an angle of $\theta_{r^{\prime }}$ with respect to the facet’s normal, and refracting towards the observer/mirror with $\theta_{edge}$. This effect is also affected by the geometry of the sample, by changing the optical path of the rays travelling in the medium (Figure S2).

In order to describe the experimental observations, we also consider the intrinsic divergence of most incident light sources and account for the distribution of angles of incidence (variable $\theta_{i}$) on the surface grating. We model our results using a Gaussian distribution of $\theta_{i}$, ranging from −20${}^{\circ }$ to 20${}^{\circ }$ (Figure 4b). Combining Equations (5)–(7), we compute the wavelength of light λ observable at $\theta_{edge}$ accounting for an incident angle $\theta_{i}$ distribution (Figure 4c). When *d* = 700 nm, light diffracts up to two diffraction orders within the medium, where the observation of higher wavelengths arises due to the contribution of the 1st order, while the lower wavelengths are due to the 2nd order. Graphically, for each fixed $\theta_{edge}$, the intersection with the $\theta_{i}$ dispersion curves yields a series of λ values. In other words, imposing $\theta_{edge}\left(\theta_{i},\lambda \right)=$ fixed value (e.g., 20${}^{\circ }$ leads to a $\lambda (\theta_{i})$ series. The intensity T(λ) of *each* ($\lambda ,\theta_{i}$) pair (sampled every 2.5${}^{\circ }$) is then assigned the corresponding Gaussian pre-factor attributed in Figure 4b, namely $g\left(\theta_{i}\right)=\exp\left(\theta_{i}^{2}/\left(2\sigma^{2}\right)\right)/\left(\sqrt{2\pi }\sigma \right)$ (with σ≃ 10, to match experimental observation). This results in the spectra shown in panel (d); the expression is discretised with 17 terms and normalised to 1. Taking $\theta_{edge}$ = 20${}^{\circ }$ for example, λ = 730 nm is expected to be observed when $\theta_{i}$ = 0${}^{\circ }$ (Figure 4c). As our model assumes this incident angle experiences a maximum, Figure 4d indicates this was associated with maximum intensity at 730 nm. Similarly, there is a minimum at λ≈ 480 nm due to the smallest weighting when $\theta_{i}$ = ±20${}^{\circ }$. Below 480 nm, the contributions of wavelengths are due to the diffraction of the 2nd order, while above this, it is contributed by the 1st order. From this model, we observe that when $\theta_{edge}$ is 20${}^{\circ }$ and 30${}^{\circ }$, the measured spectrum shows that the colour observed is red in appearance while for 40–60${}^{\circ }$, it is blue in appearance. This is in line with the experimental results, where we observe that there is a greater contribution in red at the facet at smaller $\theta_{edge}$ while at 40–60${}^{\circ }$ the facet has a more discernible blue hue to it. The profile of the modelled spectrum agrees with the experimental results in Figure 3c. The spectral signature of the incident white light source does not emit below 400 nm, and therefore, the model does not yield results below 400 nm. The measured intensities are also dependent on the geometry of the sample which affects the distance over which each ray travels; accordingly, longer ray paths lead to lower intensities recorded for their observed wavelengths (Figure S3).

**Figure 5:**
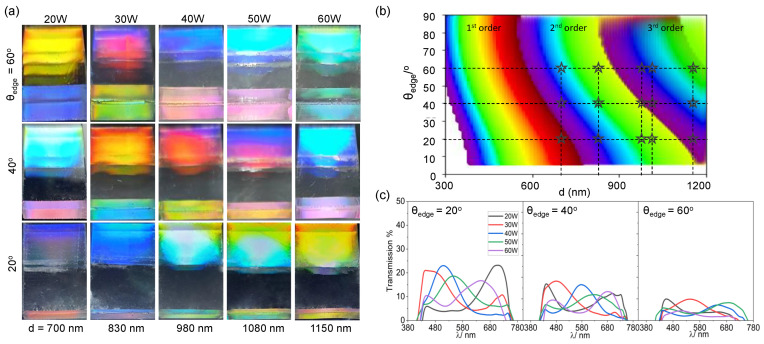
Effect of surface periodicity *d* on top (reflective diffraction) and facet (TIR) structural colour. (**a**) Optical images of illustrative samples of varying *d* (from 700 to 1150 nm) prepared through varying oxygen plasma power exposure (*P* = 20–60 W, τ = 30 s, ϵ = 0.5), acquired at $\theta_{edge}$ = 20, 40 and 60${}^{\circ }$. (**b**) Computed colour map for TIR facet colour, as a function of *d* and $\theta_{edge}$, for the first three diffraction orders. The star markers indicate conditions investigated experimentally in (**a**), showing qualitative agreement (see text). (**c**) Measured spectra of TIR facet colour, corresponding to the samples shown in (**a**) at the reference $\theta_{edge}$ = 20${}^{\circ }$, 40${}^{\circ }$ and 60${}^{\circ }$. As *P* increases, the observed colour is red-shifted and/or transitions into a higher diffraction order at a lower wavelength.

From the modelling of the structural colour, we establish that the colour observed is only dependent on the periodicity of the wrinkles and the observation angle (which themselves are affected by the incidence angle on the grating surface). We can take advantage of the tunability of plasma oxidation of PDMS to fabricate a range of structural colour designs that would achieve multifaceted structural colours. By increasing plasma oxidation power from 20 to 60 W in 10 W increments, we design wrinkles with periodicities ranging from 700 to 1150 nm. Viewing each sample at different $\theta_{edge}$ angles, 20${}^{\circ }$, 40${}^{\circ }$ and 60${}^{\circ }$, a gamut of colours is observed. The surface structural colour can be predicted based on previous work (Figure 2d) [31,47]. A similar colour map can also be used to design and predict the observed facet colour at $\theta_{edge}$ with *d*. This map was constructed from *d* = 300 to 1200 nm, which includes up to three diffraction orders, with the assumption that $\theta_{i}$ = 0${}^{\circ }$ and only taking into account the dominant colour with no colour mixing from contributions of different orders, and is shown in Figure 5b. Beyond 1.2 μm, we reach the limits in the observation of the facet colour, as the sample generates increasing numbers of diffraction orders mixing additively together (Figure S4). Overall, the model shows good agreement with the experimental results. Transmission spectra were also taken for each sample at the different angles, quantitatively showing that the structural colour is red-shifted, or shifted into higher orders, as the periodicity and $\theta_{edge}$ increases.

### 2.3. Colour Changes Induced by Environmental Conditions

**Figure 6:**
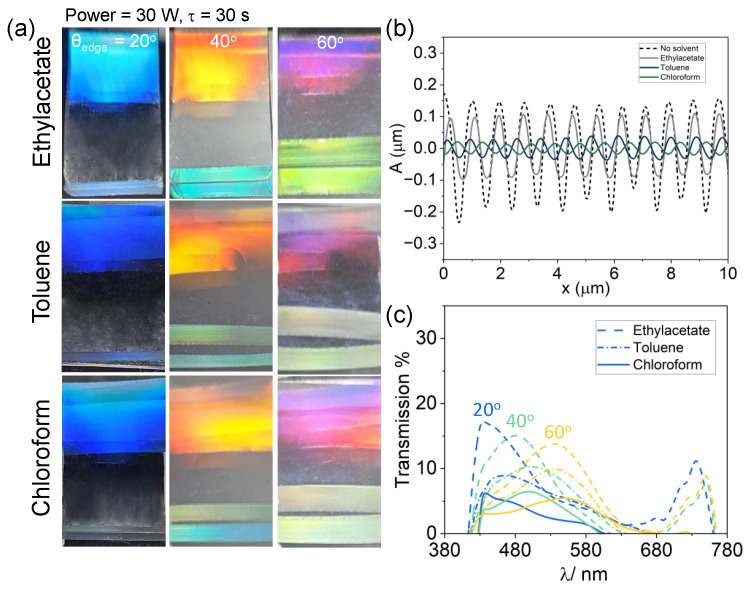
Top and facet structural colour change upon solvent-induced swelling. (**a**) Optical images of illuminated wrinkled samples with *d* = 830 nm (*P* = 30 W, τ = 30 s, ϵ = 0.5) after immersion in ethyl acetate, toluene and chloroform (in increasing order of swelling) for 10 min, at $\theta_{edge}$ = 20, 40 and 60${}^{\circ }$. (**b**) AFM line scans showing the decrease in amplitude *A*, and marginal increase in *d* upon immersion, and air drying by excess solvent removal (see text). (**c**) Measured spectra of the TIR facet colour after soaking in ethyl acetate (dash), toluene (dash–dot), chloroform (solid), at observation angles $\theta_{edge}$ = 20${}^{\circ }$ (blue), 40${}^{\circ }$ (green), 60${}^{\circ }$ (yellow). The reference dry images and spectra are shown in Figure 5a (second column) and Figure 5c.

Structural colour can reflect environmental conditions that affect the surface grating nanostructure. Environmental factors can include the presence of solvents in a liquid or gas atmosphere, or a medium with different refractive indices. When PDMS is exposed to a range of solvents, it can swell and deform significantly. Wrinkled samples (P = 30 W, τ = 30 s) were soaked in different solvents, ethyl acetate, toluene, and chloroform for 10 min each. These solvents were chosen for their different swelling ratios: 1.18, 1.31, and 1.39, respectively [48]. Prior to optical and AFM imaging, solvent-soaked PDMS coupons were carefully pad-dried with absorbent tissue paper to remove excess solvent, and allowed to air-dry for 2 min. Care was taken not to over-dry the sample and reverse swelling (as demonstrated with toluene and chloroform, for instance) [48,49,50]. The solvent-soaked samples can be seen in the optical images in Figure 6a, viewed at increasing $\theta_{edge}$ at 20, 40 and 60${}^{\circ }$. When the samples are exposed to solvents with increasing swelling ratios, the facet colours appear to be disproportionately affected, decreasing in intensity. From the AFM scans of the solvent-exposed samples in Figure 6b, we observe that the wrinkling amplitude decreases from its original to a greater extent with solvents of higher swelling ratios (from *A* ≈ 175 nm with no solvent to 12 nm with chloroform) while experiencing a marginal increase in periodicity (*d* = 830 nm to ≈900 nm). The marginal increase in periodicity results in a negligible change in structural colour on the surface, however, the decrease in amplitude causes a decrease in measured transmission intensity, shown in Figure 6c. Due to the reversibility of the swelling process [51], such systems have potential uses in sensor technology.

**Figure 7:**
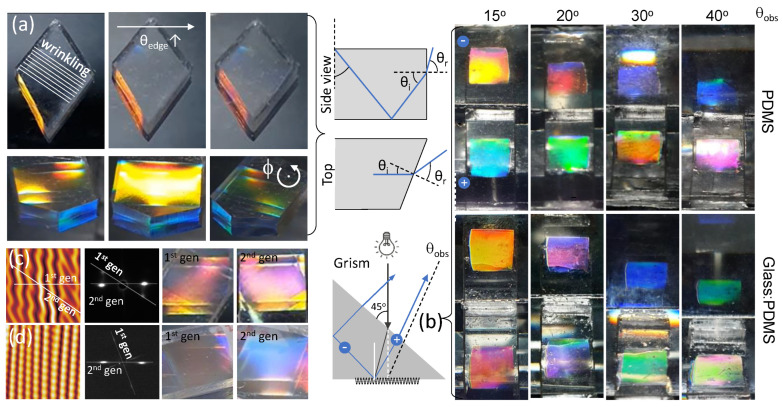
(**a**) Effect of polyhedron shape, illustrated with a diamond cut and relative orientation of the 1D wrinkled surface. The top row *d* = 620 nm (*P* = 20 W, τ = 30 s, ϵ = 0.7) depicts increasing $\theta_{edge}$, and the row below (*d* = 700 nm, *P* = 20 W, τ = 30 s, ϵ = 0.5) to varying rotation angle ϕ; the ray tracing diagrams schematically show the observations described and discussed in the text. (**b**) Experimental realisation of the coupling of a grating (on a facet) and a prism, termed “grism”, whose setup is illustrated in the sketch. Optical images obtained for PDMS (top) and glass (bottom) prisms, obtained with *d* = 980 nm (*P* = 40 W, τ = 30 s, ϵ = 0.5). Colour dispersion can be finely tuned by these two optical elements. (**c**) AFM scans of 2-dimensional (or biaxial) “sand ripple” wrinkled surfaces, fabricated by two equal mechanical wrinkling steps with ϵ = 0.2, *P* = 50 W, τ = 30 s, at an angle of ϕ = 30${}^{\circ }$ between the two generations; (**d**) 2D pattern generated with 1st generation $\epsilon_{1}$ = 0.5, *P* = 50 W, τ = 30 s, and 2nd generation $\epsilon_{2}$ = 0.2, *P* = 20 W, τ = 30 s, with ϕ = 70${}^{\circ }$ between generations.

Specimen geometry can be exploited to further expand the variation of multifaceted structural colours. To date, results for 1D samples prepared using a rectangular slab were presented, resulting in facet colour to be observed in the same direction as that from the wrinkled surface. From such samples, the edges can be subsequently cut at different angles, to examine the impact of geometry. Cutting is carried out only after the fabrication of wrinkles to prevent any inhomogeneities in strain application. A schematic of the side and top view shows the expected direction travel of light rays while exiting a sample as shown in Figure 7a. In a rectangular sample, we only need to consider the side view to determine the pathway of ray travel. As previously shown, we can determine the $\theta_{edge}$ for observed wavelengths, and the facet colours are observed in the same direction as the surface. However, in a cut sample, we also have to consider another dimension for light refraction, where the direction of observation of facet colour is offset from that of the surface colour.

To demonstrate this effect, Figure 7a shows two samples with “diamond” cuts at the edges, the top row sample prepared at *P* = 20 W, τ = 30 s, ϵ = 0.7 with periodicity *d* = 620 nm, while the bottom at *P* = 20 W, τ = 30 s, ϵ = 0.5 with *d* = 700 nm. The sample with periodicity *d* = 620 nm was observed with increasing $\theta_{edge}$ at an angle ϕ = 40${}^{\circ }$ offset, where ϕ is defined as the angle about the sample’s horizontal axis (Figure 1a), causing the wrinkling direction to be non-perpendicular to the observer. Since uniaxial wrinkled surfaces act as 1D phase gratings [52], these diffract light solely in the direction perpendicular to that of the orientation of the wrinkles. As a result, structural colour is only observable along that direction, and within a narrow off-specular range (of approximately Δϕ± 5–10${}^{\circ }$, associated with wrinkling disorder and finite illuminated spot size). Structural colour is otherwise not observable away from this diffraction plane, as illustrated in Figure 7a where only edge colour, due to facet TIR, is visible. As with the other samples, the colour of the facet can be seen to change with increasing $\theta_{edge}$, where it transitions from yellow to red. In the 2nd sample, where *d* = 700 nm, the sample was observed at a fixed $\theta_{edge}$ = 50${}^{\circ }$, and rotated about ϕ. We observe that the surface colour yellow can be effectively ‘switched on or off’ along with the facet colours. By exploiting different directions of cuts in the samples, we can manipulate different variations and combinations in the multi-faceted structural colours of these samples.

Incorporating surface grating with a prism, we demonstrated the fabrication of GRISMs (Figure S5). In this context, a GRISM is a compound optical element that significantly reduces the influence of light dispersion from the individual elements. A prism deflects violet light more than red, while a diffraction grating deflects red more than violet. By combining the two, light can be separated into its components while offsetting the beam deviations resulting from each element. In a simple realisation of a GRISM, we placed model prisms made of either PDMS (*n* = 1.41) or glass (*n* = 1.52) on top of a wrinkled PDMS sample (*P* = 40 W, *d* = 980 nm) to create GRISMs (PDMS, and Glass:PDMS) as shown in the schematic in Figure 7b. The samples were placed such that the wrinkled surface was in contact with the prism. Employing white light, the GRISMs were viewed at increasing $\theta_{obs}$, from 15${}^{\circ }$ to 40${}^{\circ }$. $\theta_{obs}$ is taken from the normal of the base of the GRISM, directly under the centre of the incident light.

GRISMs show a “dual” image of the wrinkled surface exhibiting different colours. When white light is shone vertically, it illuminates the prism surface at an angle of 45${}^{\circ }$, and then refracts into the medium, thus impinging on the grating at an angle, before diffracting back into the medium. The grating diffracts positive and negative diffraction orders, denoted in the schematic as + and −. The rays from the positive orders are then incident on the top face of the GRISM and are refracted out, while those from the negative orders will first hit the side face of the grism. If the angle of the rays incident on the side face exceeds the critical angle, it undergoes TIR before exiting from the top. The combination of the two diffraction orders produces dual-coloured appearances, shown in the optical images. From an observer’s perspective in an optical image, the top colour is produced by the negative diffraction order while the bottom is from the positive order. As $\theta_{obs}$ increases, we observe a gamut of different colours, which can also be manipulated by changing the material composition of the prism. Figure 7b shows that by changing the refractive index of the medium (PDMS to glass), we are able to obtain distinct colours in the GRISMs.

Finally, building on our previous work, we explore the manipulation of structural colour in two-dimensional (2D) samples further [31,53,54]. 2D samples can either be fabricated in a sequential or simultaneous wrinkling step. Here, the 2D samples are fabricated through the superposition of wrinkles in a sequential step (details described in the Section 3). By manipulating the conditions in the first and second steps, we achieve different intensities and/or colours depending on the orientation of the sample. Figure 7c illustrates a specimen fabricated with two equal mechanical wrinkling steps with ϵ = 0.2, *P* = 50 W, τ = 30 s, at an angle of ϕ = 30${}^{\circ }$ between the 2 wrinkling generations. The resulting structure shows a ‘sand ripple’ pattern, producing a diffraction grating where two sets of diffraction orders can be observed. In the light scattering image, the 1st generation diffraction appears at ϕ = 30${}^{\circ }$ from the horizontal with an associated wrinkle wavelength of $d_{1}$, while the 2nd generation diffraction pattern is along the horizontal (*x*) axis with $d_{2}$. As the conditions of the two generations are equal, $d_{1}$ = $d_{2}$ and the structural colour observed by the two generations at a given $\theta_{edge}$ are similar. However, in the superposition of wrinkles, the 2nd generation suppresses the 1st generation’s amplitude, causing the intensity of the 2nd generation to be greater than that of the 1st generation.

As the angle between the generations, ϕ increases, the 2nd generation dominates. This is the result of the relationship between the formation of the surface topography and its principal coordinates *x* and *y*. When the second generation has a larger strain component in the *y* direction compared to the *x* direction, it suppresses the amplitude in the first generation to a greater extent. Figure 7d shows a 2D sample fabricated under two different conditions: 1st generation $\epsilon_{1}$ = 0.5, *P* = 50 W, τ = 30 s, and 2nd generation $\epsilon_{2}$ = 0.2, *P* = 20 W, τ = 30 s, with ϕ = 70${}^{\circ }$ between generations. In order to observe any structural colour in the 1st generation at high ϕ, a large mismatch in strain and plasma conditions is required to compensate for the decrease in amplitude in the 1st generation. This mismatch in conditions results in different colours being observed in the generations, with a low-intensity red hue in the 1st generation, and a bright blue observed in the 2nd generation.

## 3. Materials and Methods

PDMS (Sylgard 184, Dow Corning, Midland, MI, USA) coupons were prepared by casting a mixture of prepolymer and curing agent with a mass ratio of 10:1. The liquid mixture was stirred vigorously, degassed under vacuum, deposited onto a glass plate and cured at 75 ${}^{\circ }$C in a convection oven for 1 h to crosslink into a PDMS elastomer slab with the required thickness (ranging from 2.0 to 3.0 mm). The coupons of 1.5 cm in width and varying lengths (4–8 cm) were then cut with a blade.

In order to create a bilayer with a glassy skin, surface plasma oxidation of the PDMS coupon samples was performed with a 40 kHz Diener Plasma (Femto, Diener Electronic, Ebhausen, Germany), fitted with a pressure sensor (TM 101, Thermovac, Leybold GmbH, Cologne, Germany) and connected to oxygen (BOC, 99.5%). Samples were treated under plasma at 10 W intervals from *P* = 20 to 60 W, with exposure time kept constant at τ = 30 s. The chambers were evacuated to a pressure of 0.1 mbar, before introducing oxygen for 5 min until the pressure reached 0.2 mbar and stabilised. The plasma was then ignited, at the required power and exposure time.

One-dimensional (1D), regular, sinusoidal patterns were fabricated by imposing uniaxial strain on a PDMS coupon (typically 2.5 cm long × 1.5 cm wide) using a strain stage. The strain clamps were placed 1 cm apart onto the PDMS coupon, and the samples were stretched to a prestrain of 0.5, before undergoing plasma oxidation, and subsequently released from strain, yielding a sinusoidal diffraction grating (1 cm long) of prescribed wavelength and amplitude. The prestrain is calculated with respect to the initial ($L_{0}$) and final distance ($L_{1}$) between the clamps, $\varepsilon =\frac{L_{1}-L_{0}}{L_{0}}$.

Two dimensional (2D) surfaces were fabricated by a wave superposition method, reported previously [36,53,54]. In short, an initial 1D sample is fabricated and then replicated onto fresh PDMS. The “replica” is generated by first coating the “master” with octadecyl trichlorosilane (OTS) (Acros Organics, 95%) from the vapour phase, and then casting liquid PDMS, which is then crosslinked at 75 ${}^{\circ }$C for 1 h and peeled off from the master. This process offers excellent replication fidelity. The replica is then tilted so that the 1D pattern is oriented along the desired ϕ angle and cropped in a rectangular shape, to avoid inhomogeneities during the secondary strain application ($\epsilon_{2}$) and plasma oxidation step (with independently variable parameters of ε, *P* and τ). Once the strain is released a 2D secondary pattern is formed.

The surface topographies were characterised by atomic force microscopy (AFM) using a Bruker Innova microscope, in tapping mode at 0.2 Hz, equipped with Al-coated Si tips (MPP-11100-W, Bruker, Billerica, MA, USA) and analysed with the in-built Nanoscope software. Structural colour spectra were recorded using BLACK-Comet UV-VIS Spectrometer (StellarNet Inc, Tampa, FL, USA) with F600-VIS-NIR fiber optic cable with a white light source (Advanced Illumination, Rochester, VT, USA) in a dark environment. Optical photos were taken with a digital camera.

## 4. Conclusions

In this paper, we demonstrate the design and fabrication of multi-faceted structural colour on a transparent elastomeric material (PDMS), by patterning surface diffraction grating via oxygen plasma oxidation and exploiting TIR. Tunable wrinkles on the surface yield striking structural color by acting as reflective diffraction gratings, whose response we model and validate experimentally. The main novelty of our paper is the exploration of TIR as a means to generate structural colour on the other facets of a 3D material, emulating a range of naturally-occurring materials. TIR selects a limited subset of diffracted colours which are propagated, while the rest are refracted. The “side” (facet) colour can thus differ from the surface-diffracted colour and can be controlled by well-defined design rules which we establish and describe in this work. The design and selection of sample geometry can pave the way for an extensive library of designs, with “gem cut-like” characteristics. Macroscopically shaped objects (such as cuboids, triangles, etc.) thus affect light propagation and colour appearance, and we generate a so-called GRISM for the illustration of an optical device. An elastomer colour sensor is also demonstrated since elastomeric materials are sensitive to some gaseous or liquid medium changes. The design parameter space for inducing “multifaceted” colour by coupling diffraction and TIR is very large and promising. Our findings are expected to be relevant to a wide range of applications, including displays, packaging, and sensors.

## Data Availability

Data is contained within the article or supplementary material and available from the authors upon request.

## Supplementary Materials

The following supporting information can be downloaded at: https://www.mdpi.com/article/10.3390/molecules28041710/s1, Figure S1. Light source spectrum and PDMS optical attenuation; Figure S2. Effect of sample geometry (thickness); Figure S3. Effect of sample geometry (length); Figure S4. Limits of facet TIR; Figure S5. Fabrication of an illustrative GRISM.

## Sample Availability

Samples of the compounds are available from the authors upon request.
