# Supplementary material for: Multifaceted Structurally Coloured Materials: Diffraction and Total Internal Reflection (TIR) from Nanoscale Surface Wrinkling
Source: Molecules. 2023 Feb 10;28(4):1710. doi: 10.3390/molecules28041710 (PMC9962751; doi:10.3390/molecules28041710)
Supplement: Supplementary file 1 [file molecules-28-01710-s001.zip › molecules-2197861-supplementary.pdf]

Supplementary Information: Multifaceted structurally coloured materials: diffraction and total internal reflection (TIR) from nanoscale surface wrinkling

Annabelle Tan,[1,2] Zain Ahmad,[1] Pete Vukusic [3] and João T. Cabral[1,2,*]

[1] *Department of Chemical Engineering, Imperial College London, London SW7 2AZ, UK*

[2] *Centre for Processable Electronics, Imperial College London, London SW7 2AZ, UK*

[3] *School of Physics, University of Exeter, Stocker Road, Exeter EX4 4QL, UK*

E-mail: j.cabral@imperial.ac.uk

1 Light source spectrum and PDMS optical attenuation

The spectrum of our light source is presented in Fig. S1(a). The optical attenuation of PDMS was measured using a range of PDMS slabs with varying thicknesses, up to 12 cm. Optical transmission data are shown in Figure S1(b). The transmission was then computed as $T = (I_{sample} - I_{background})/I_{ref} - I_{background})$, where I_{ref} corresponds to the reference spectrum and $I_{background}$ is the background spectrum accounted for by the spectrometer for the environmental background. The calibration of the spectrometer with the PDMS system yields $T = e^{-0.021h}$, where h is the PDMS thickness, as shown in Figure S1c).

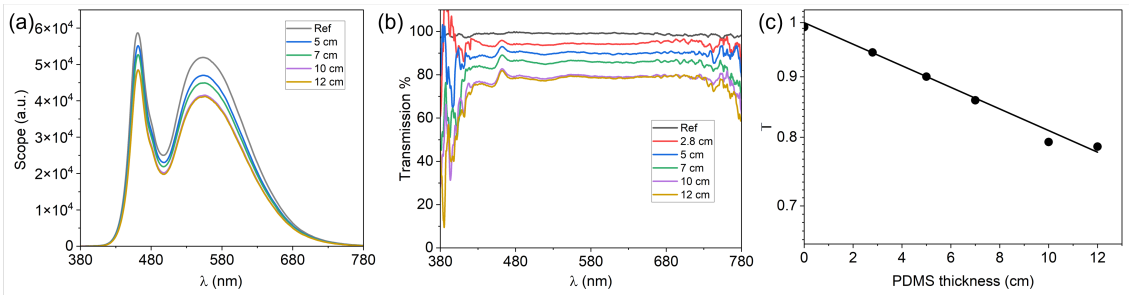

Figure S1 Spectrometer measurements with different thicknesses of PDMS: (a) Measurement intensity spectra; (b) Computed transmission data as a function of PDMS thickness; (c) Calibrated transmission dependence on thickness, yielding $T = e^{-0.021h}$, where h is PDMS thickness.

2 Effect of sample geometry

The geometry of the sample can affect the perceived structural colour from the facet. As discussed in the main paper, when the TIR rays exit the PDMS at the facet, they can either exit towards the observer (upwards) or towards the substrate (downwards). We can capture the latter signal by employing a reflective surface, for instance, a mirror. Radiation exiting towards the mirror can be observed in reflection, a nd i ts colour spectrum can be analysed. Employing reference conditions (P = 20 W, τ = 30 s, ε = 0.5), samples of two slightly different thicknesses (h = 2.75 mm and 3 mm) were prepared, keeping the length constant where L_{edge} = 1.5 cm. From Figure S2a, we observe that at θ_{edge} = 30° and 40°, there is a slight difference in the optical images between h = 2.75 mm and 3 mm, where the facet colour appearing from the facet and the mirror 'reflection' is apparently switched between the two thicknesses. This is a geometric effect, that can be readily explained by ray tracing, shown in Figure S2b. At these two θ_{edge}, we find the spectra in Figure S2c have a maximum at ≈ 750 nm, which is responsible for the contribution in red colour. The single ray tracing diagram from the centre of the diffraction grating surface illustrates the observation: When h = 2.75 mm, the ray exits downwards, towards the reflective surface, and a red-dominant component is observed through the mirror. Conversely when h = 3 mm, the depicted ray exits upwards, towards the observer, and a dominant red colour is then observed at the facet. Figure S2c shows the associated spectra relating to the samples at the various θ_{edge}. The shape of the spectra is similar to that of samples of the same condition, showing that the colours observed are dependent on the wrinkle periodicities. The intensity of the thicker sample is lower than that of the thinner ones, due to the longer pathlength and associated light attenuation.

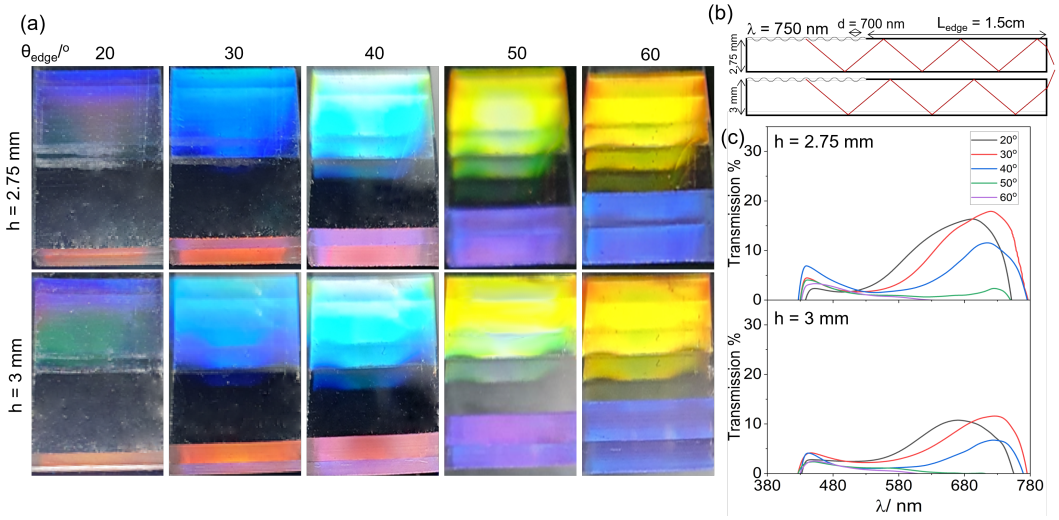

Figure S2 (a) Optical images of structural colour of a 1D surface wrinkled sample with d = 700 nm (P = 20 W, τ = 30 s, ε=0.5), with L_{edge} = 1.5 cm and different thicknesses h = 2.75 mm and 3 mm, observed with increasing θ_{edge}. (b) Ray tracing diagram of a 750 nm ray for both sample thicknesses, depicting the different direction of ray exit at the facet. (c) Recorded transmission spectra for each sample at corresponding θ_{edge}.

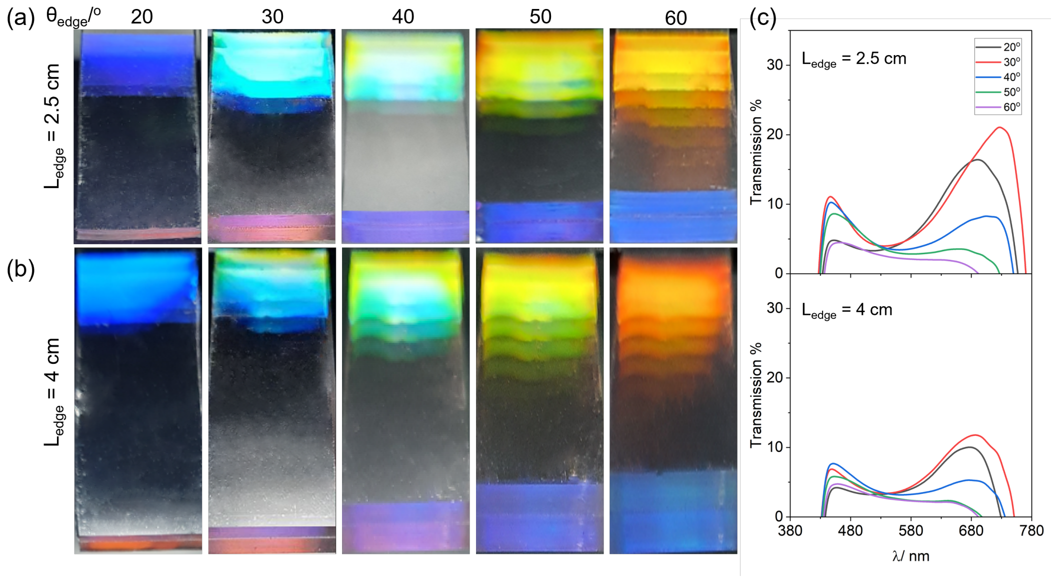

Figure S3 Optical images of the structural colour of wrinkled samples with d = 700 nm and h = 2.1 mm, with lengths (a) L_{edge} = 2.5 cm, (b) L_{edge} = 4 cm. (c) Measured spectra for samples with the different L_{edge} as a functio of observation angle.

Specimens of different lengths were also prepared under otherwise the same conditions as those of Fig. 3 of the main paper, and Figure S2, namely with ε = 0.5, P = 20 W, τ = 30 s, where L_{edge} = 2.5 cm and 4 cm (1.5 cm reported in the main paper). Upon clamping the PDMS coupons with different lengths, scotch tape was used to cover up the exposed areas, ensuring that the edge portion did not undergo plasma treatment. This experiment serves to show the feasibility of manipulating the geometry of PDMS coupons to accommodate longer samples. As in previous experiments, Figure S3 shows the structural colours of the sample, surface and facet when θ_{edge} varies from 20 to 60°. For these geometries, the facet colours are similar, only decreasing in intensity with increasing length (and thus light attenuation along the pathlength, and losses at each surface reflection). The structural colour of the surface differs slightly due to the offset in angle $\theta_{DG'}$, affected by L_{edge}.

3 Limits of facet TIR

A series of samples with higher periodicities were fabricated with plasma conditions P = 20 W, ε = 0.2, at increasing exposure times from 90 to 240 s. These conditions were selected as a lower prestrain allows higher periodicities to be achieved. With increasing periodicities, facet TIR colour becomes progressively less visible. At 1.2 μm, a faded purple hue can be observed at θ_{edge} = 20°, while the other conditions, up to 2μm, display no observable facet colour. As d increases, additional orders diffract within the medium, causing wavelengths from different orders to undergo mixing. In addition, wavelengths from higher diffraction orders also exhibit lower intensities. As a result of the combination of both effects, we conclude that facet TIR can be prominently observed when wrinkle surfaces are fabricated at the nano-scale.

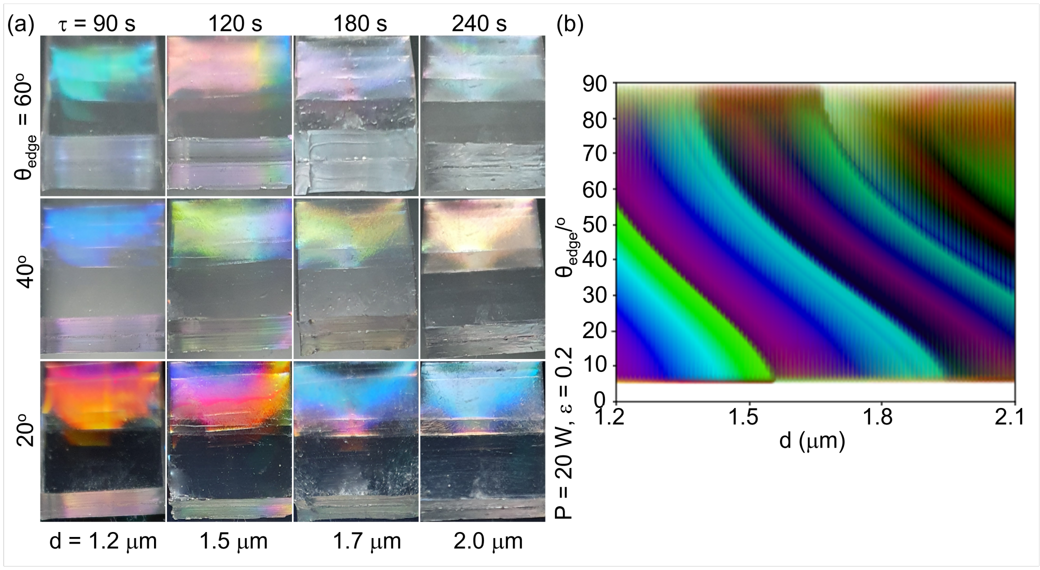

Figure S4 (a) Optical images of samples fabricated at P = 20 W, ε = 0.2, and exposure times from τ = 90 to 240 s. These samples show the decreasing manifestation of facet TIR colours with increasing d. (b) Colour map of facet TIR for d = 1.2 to 2.1 μm, showing the mixing of up to 6 diffraction orders.

4 Fabrication of an illustrative GRISM

GRISMS are fabricated through the incorporation of diffraction grating onto the surface of a prism. Here, we demonstrate the fabrication of a GRISM structure by placing an isosceles, right-angle prism, atop a wrinkled surface, with the wrinkles in contact with the prism, shown in Figure S5a. We use two materials for the prims: glass and PDMS. The setup with glass prism (Edmund Optics) can be seen in Figure S5b, with length and width of 3 cm each. The PDMS prism was fabricated using a PMMA mould, made by laser cutting and solvent bonding, as shown in Figure S5c. Fresh PDMS was then poured into the mould and cured, and the PMMA mould was unmounted to yield a PDMS prism with identical dimensions as that made of glass.

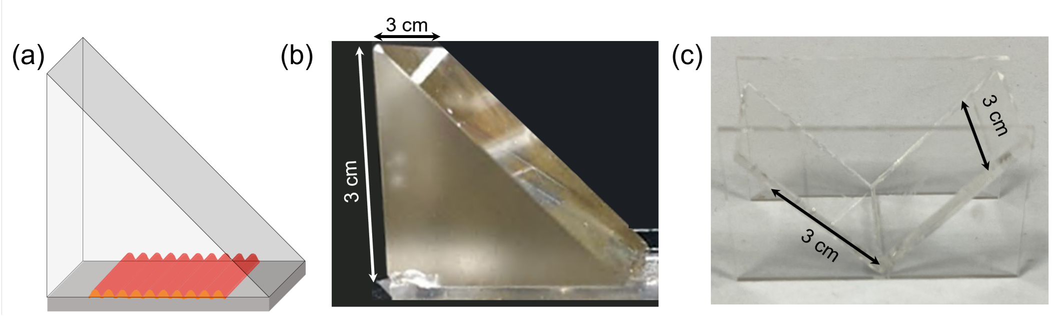

Figure S5 (a) Illustration of the fabrication of a 'GRISM' by combining a prism and a wrinkled PDMS layer onto its side surface. (b) Isosceles right-angled glass prism, with side length of 3 cm and width of 3 cm, coupled with a wrinkled surface, to demonstrate the dispersion of colours on the two surfaces of the prism. (c) A PMMA mould is utilised to create a PDMS prism. The mould is obtained by joining individual PMMA laser-cut pieces of thickness 3 mm.
